# Flat Inferior Vena Cava on Computed Tomography for Predicting Shock and Mortality in Trauma: A Meta-Analysis

**DOI:** 10.3390/diagnostics12122972

**Published:** 2022-11-28

**Authors:** Do Wan Kim, Hee Seon Yoo, Wu Seong Kang

**Affiliations:** 1 Department of Thoracic and Cardiovascular Surgery, Chonnam National University Hospital, Chonnam National University Medical School, Gwangju 61469, Republic of Korea; 2 Department of Surgery, Cheju Halla General Hospital, Jeju 63127, Republic of Korea; 3 Department of Trauma Surgery, Jeju Regional Trauma Center, Cheju Halla General Hospital, Jeju 63127, Republic of Korea

**Keywords:** trauma, inferior vena cava, hypovolemia, shock, computed tomography

## Abstract

Hypovolemia may be underestimated due to compensatory mechanisms. In this systematic review and meta-analysis, we investigated the diagnostic accuracy of a flat inferior vena cava (IVC) on computed tomography (CT) for predicting the development of shock and mortality in trauma patients. Relevant studies were obtained by searching PubMed, EMBASE, and Cochrane databases (articles up to 16 September 2022). The number of 2-by-2 contingency tables for the index test were collected. We adopted the Bayesian bivariate random-effects meta-analysis model. Twelve studies comprising a total of 1706 patients were included. The flat IVC on CT showed 0.46 pooled sensitivity (95% credible interval [CrI] 0.32–0.63), 0.87 pooled specificity (95% CrI 0.78–0.94), and 0.78 pooled AUC (95% CrI 0.58–0.93) for the development of shock. The flat IVC for mortality showed 0.48 pooled sensitivity (95% CrI 0.21–0.94), 0.70 pooled specificity (95% CrI 0.47–0.88), and 0.60 pooled AUC (95% CrI 0.26–0.89). Regarding the development of shock, flat IVC provided acceptable accuracy with high specificity. Regarding in-hospital mortality, the flat IVC showed poor accuracy. However, these results should be interpreted with caution due to the high risk of bias and substantial heterogeneity in some included studies.

## 1. Introduction

Hypovolemia is crucial in the diagnosis and treatment of trauma patients [1] and is one of the most common causes of preventable death in trauma patients and accompanies hemorrhagic shock [2]. However, stable vitals do not directly correlate with a negative hypovolemia diagnosis. According to the clinical guideline by the Advanced Trauma Life Support (ATLS), vitals, such as blood pressure or heart rate, may be stable even though with substantial blood loss in internal organs [3]. To achieve effective damage control resuscitation, early detection of hypovolemia is crucial and several prediction models, such as the Assessment of Blood Consumption (ABC) score or the Trauma-Associated Severe Hemorrhage (TASH) score, have been introduced [4]. However, the accuracy of these prediction models varies from a 0.51 area under the receiver operating curve (AUC) to a 0.97 AUC according to various clinical settings [4].

These scoring systems are generally based on point-of-care ultrasounds, which detect free fluid on the abdomen or pelvic cavity. In our previous systematic review and meta-analysis, ultrasounds measuring the respiratory variation of the inferior vena cava (IVC) were shown to accurately predict volume responsibility with a pooled AUC of 0.86 [5]. In addition, variation of IVC diameter has been shown to predict volume status in many previous studies [5]. However, ultrasound-guided measuring depends on the practitioner’s skill or experience. In contrast, computed tomography (CT) can provide objective measurement of the IVC regardless of the operator. Modern multi-detector computed tomography (MDCT) showed good discrimination power regarding internal bleeding in a previous meta-analysis [6]. Although only patients with vital stability can undergo CT, the CT can provide critical information from initially stable patients. A recent systematic review including 23 studies reported signs of post-traumatic hypovolemia on abdominal CT [7]. The authors reviewed components of the hypovolemic shock complex, such as a flat IVC, IVC halo, aortic diameter, shock bowel, pancreas enhancement, peripancreatic fluid, and adrenal enhancement [7]. However, they did not report quantitative pooling results [7]. This previous systematic review included studies published through July 2018. Here, in the present study, we tried to update the searching and conducted a meta-analysis to calculate pooled estimates.

This systematic review and meta-analysis investigated the diagnostic accuracy of a flat IVC on CT for predicting the development of shock and mortality in trauma patients. We focused on flat IVCs instead of other components of the hypovolemic complex.

## 2. Materials and Methods

### 2.1. Published Study Search and Selection Criteria

This study was performed according to the Preferred Reporting Items for Systematic Reviews and Meta-Analysis of Diagnostic Test Accuracy (PRISMA-DTA) search and selection criteria [8]. The preset protocol of this study was registered on PROSPERO (CRD42022325000, https://www.crd.york.ac.uk/prospero/ accessed on 5 May 2022). Relevant articles were obtained by searching the title and abstract in PubMed, EMBASE, and Cochrane databases through 16 September 2022. These databases were searched using the following keywords: ((“inferior vena cava”) OR (IVC)) AND (diameter OR collapsibility OR variation OR variability OR measurement OR flatness OR flat OR flattened OR ratio) AND (trauma OR traumatic OR hypovolemic OR hypovolemia) AND ((“computed tomography”) OR (CT)). In addition, we manually searched the reference lists of relevant articles. We screenedthe titles and abstracts of all searched articles for exclusion. We screended review articles and previous meta-analysesto obtain additional eligible studies. We reviewed the search results, and articles were included if the study investigated flat IVCs on CT to predict the development of shock or mortality.

The primary outcome of this systematic review was the diagnostic test accuracy (DTA) of a flat IVC for the development of shock after initial CT scans in trauma patients. The secondary outcome was the DTA of a flat IVC for in-hospital mortality.

The inclusion criteria for this review were as follows: (1) the study population included trauma patients; (2) a measurement of a flat IVC using IVC ratio or IVC diameter was performed as an index test; (3) after CT scan, the development of shock and in-hospital mortality were detected; (4) adequate information was provided to compute the DTA and construct a 2-by-2 contingency table consisting of true positive (TP), false positive (FP), false negative (FN), and true negative (TN) outcomes. We exclude articles that involved another disease (non-trauma), those that did not include 2-by-2 contingency table information, non-original articles, non-human studies, or those published in a language other than English.

### 2.2. Data Extraction

Two investigators extracted data from all eligible studies. Extracted data from each of the eligible studies included the author’s name, year of publication, study location, study design and period, number of patients analyzed, index tests, threshold of index tests, measured site of IVC, reference standard, CT modality (slice), and vitals during CT scan. The number of TPs, FPs, FNs, and TNs from the index test in predicting shock or mortality were collected. When the number of 2-by-2 contingency tables was not reported directly, we calculated the number of TPs, FPs, FNs, and TNs using the total number, prevalence, sensitivity, specificity, negative predictive value, and positive predictive value.

### 2.3. Quality Assessment

Two investigators independently reviewed all studies. Disagreements regarding the study selection and data extraction were resolved by a consensus. As recommended by the Cochrane Collaboration, the Quality Assessment of Diagnostic Accuracy Studies (QUADAS)-2 tool was used to evaluate the risk of bias in the diagnostic test accuracy [9]. Disagreements after using the QUADAS-2 tool were resolved by discussion with a third independent author. The QUADAS-2 assesses four domains for bias and applicability as follows: (1) patient selection, (2) index test, (3) reference standard, (4) flow and timing.

### 2.4. Statistical Analysis

We constructed a 2-by-2 contingency table (TP, FP, FN, TN) by calculating or extracting data from each primary study. We used the Bayesian inference model because the frequentist method may be statistically unstable when the number of eligible studies is small (<20) [10]. We calculated the pooled sensitivity, specificity, positive likelihood ratio, negative likelihood ratio, diagnostic odds ratio, and AUC with 95% credible intervals (CrIs) using the Bayesian bivariate random-effect meta-analysis model [10]. We implemented the Bayesian meta-analysis using non-informative priors. We also used the hierarchical summary receiver operating characteristics (HSROC) model [11]. An AUC close to 1 and 0.5 indicated a strong and poor test, respectively. An AUC of 0.7 to 0.8 is considered acceptable, 0.8 to 0.9 excellent, and more than 0.9 outstanding in general [12]. To calculate the area under the summary receiver operating characteristics (SROC) curve, we used the Rutter and Gatsonis’s SROC curve [11]. I2 was calculated from the results as I2 = 100% × (Q − df)/Q, where Q is Cochran’s heterogeneity statistic and df is the degrees of freedom to investigate the heterogeneity [13]. Results with *p*-values < 0.05 were considered statistically significant. I2 lies between 0% and 100%. A value of 0% indicates no observed heterogeneity, and values greater than 50% are considered to indicate substantial heterogeneity. Spearman’s correlation coefficient between sensitivity and specificity was calculated after logit transformation to detect the threshold effect. We firstly assessed publication bias visually using a scatter plot. We used the diagnostic log odds ratio (lnDOR), which has a symmetrical funnel shape when publication bias is absent [14]. We conducted formal testing for publication bias by the regression of lnDOR against the square root of the effective sample size, with *p* < 0.05 for the slope coefficient indicating significant asymmetry [14]. Subgroup and sensitivity analyses were conducted to investigate the heterogeneity across the eligible studies. We used the R programming language, version 4.1.2 (R foundation, Vienna, Austria), with “meta4diag” package for the Bayesian statistical analysis. The “mada” package for frequentist statistics was used for I2 calculation. QUADAS-2 assessment was performed using Review Manager Software 5.4 (The Cochrane Collaboration, Oxford, Copenhagen, Denmark). Deek’s funnel plot for publication bias was performed using STATA version 17.0 (Stata Corporation, College Station, TX, USA).

## 3. Results

### 3.1. Selection and Characteristics of Included Studies

**Figure 1:**
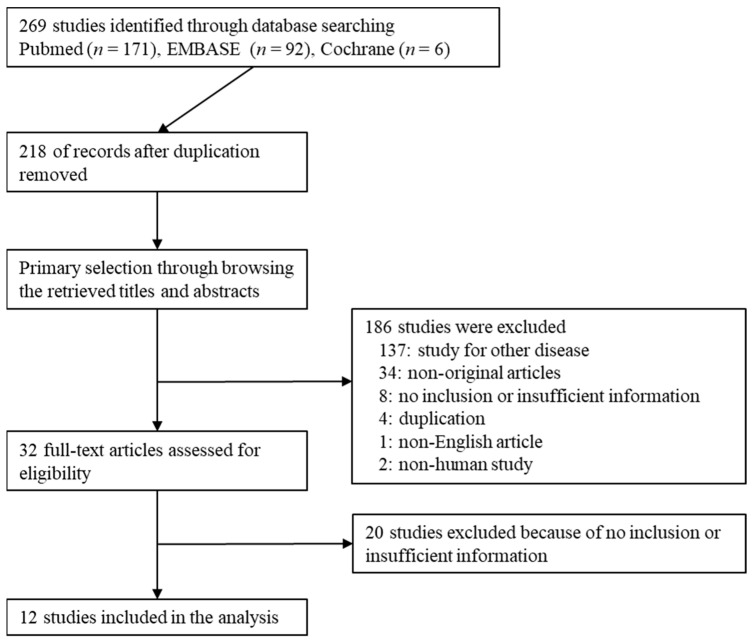
Flow diagram for identification of eligible studies.

A total of 269 studies were identified by searching the databases. After removing duplicates, 218 studies were retrieved. We excluded 186 studies by reviewing the titles and abstracts because they included other diseases (*n* = 137), were non-original (*n* = 34), did not include insufficient information (*n* = 8), were duplicates (*n* = 4), were written in a non-English language (*n* = 1), or were non-human studies (*n* = 2). We reviewed 32 full-text articles. After the full-text review, 20 articles were excluded due to no inclusion (*n* = 5) or insufficient information (*n* = 15). Finally, 12 studies [15,16,17,18,19,20,21,22,23,24,25,26] comprising a total of 1706 patients were included in this review (Figure 1). Detailed information about the eligible studies is shown in Table 1.

All 12 included studies were summarized in Table 1. Nine studies reported the development of shock [15,18,19,20,21,22,23,24,26], and eight reported in-hospital morality [15,16,17,19,20,23,24,25]. All studies reported flat IVCs as an index test. In the included studies, a flat IVC was defined using the ratio of transverse/anteroposterior diameter (T/AP) or anteroposterior (AP) diameter. The T/AP ratio was used in nine studies [16,18,19,20,21,22,23,24,26], while just the AP diameter was used in the other two studies [17,25]. In four studies [15,19,21,26], a flat IVC was measured at the infra-hepatic IVC level. In seven studies [16,18,20,22,23,24,25], a flat IVC was measured at the renal vein level. In seven studies [18,19,20,22,23,24,25], a CT scan was performed within one hour from admission. In six studies [16,18,19,20,21,24], vitals during the CT scan were stable, while the other six studies [15,17,22,23,25,26] did not report vitals during the CT scan. In six studies [18,21,22,23,24,26], MDCT over 16 slices was performed.

### 3.2. Quality Assessment

**Figure 2:**
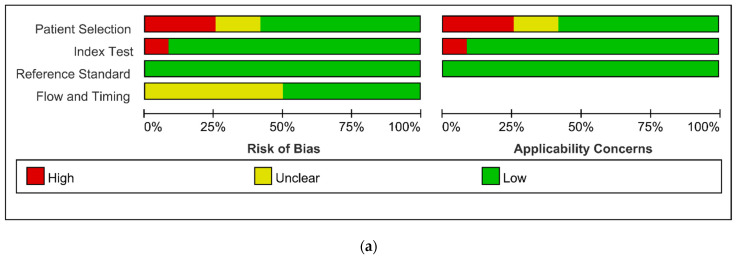

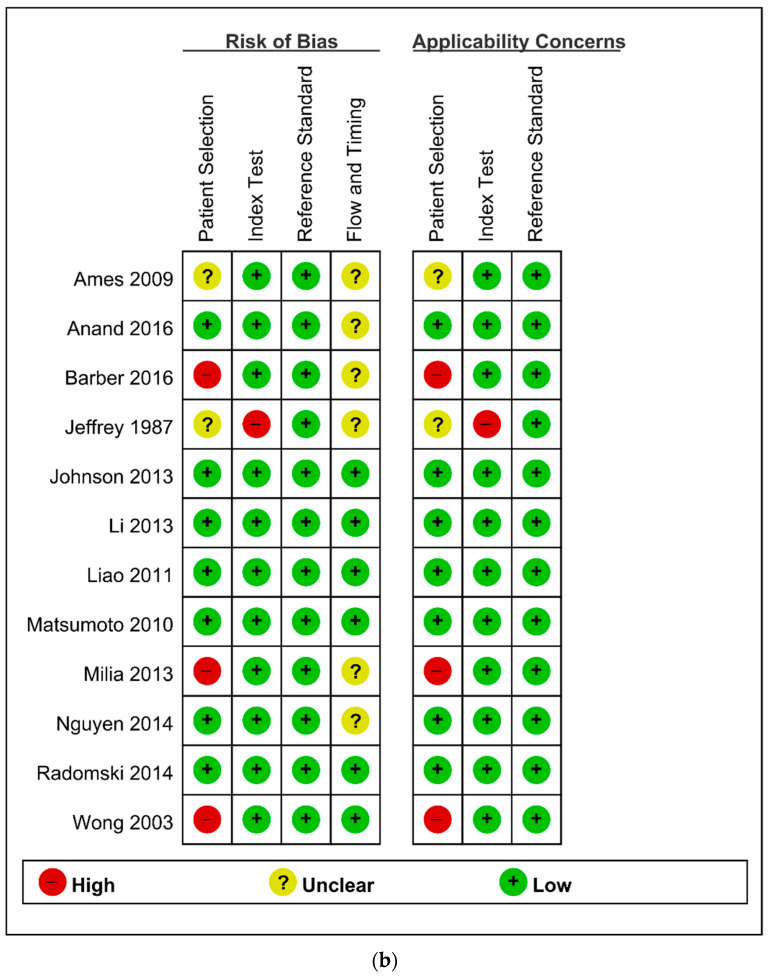
Risk of bias and applicability concerns graph (**a**) and summary (**b**): review authors’ judgements about each domain presented as percentages across included studies [15,16,17,22,23,25,26].

All studies included in this study were observational. The details of the quality assessment are depicted in Figure 2. In the reviewer’s judgement, three studies [16,22,26] had a high risk of bias and high concerns regarding applicability in terms of patient selection. Wong et al. [16] included blunt trauma with contrast extravasation. Milia et al. [22] included only elderly (≥ 55 years) patients. Barber et al. [26] included only pediatric patients. One study [15] had a high risk of bias and high concerns regarding applicability in terms of index tests, where Jeffrey et al. [15] did not report the threshold or measuring method of a flat IVC. Two studies [15,17] had an unclear risk of bias in terms of patien selection. Six studies [15,17,22,23,25,26] had an unclear risk of bias in terms of flow and timing.

### 3.3. DTA Review

**Figure 3:**
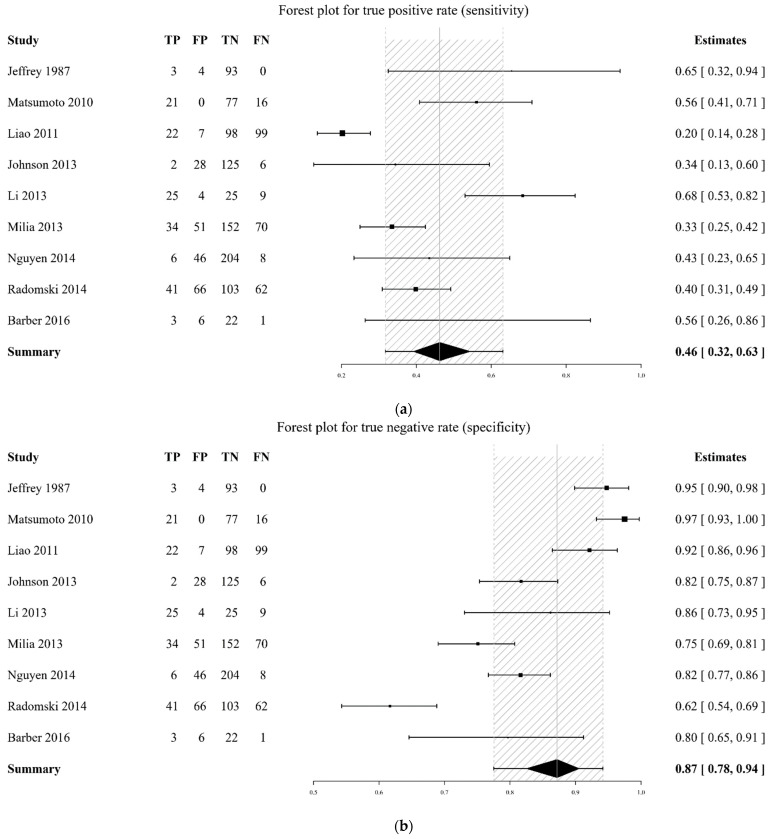

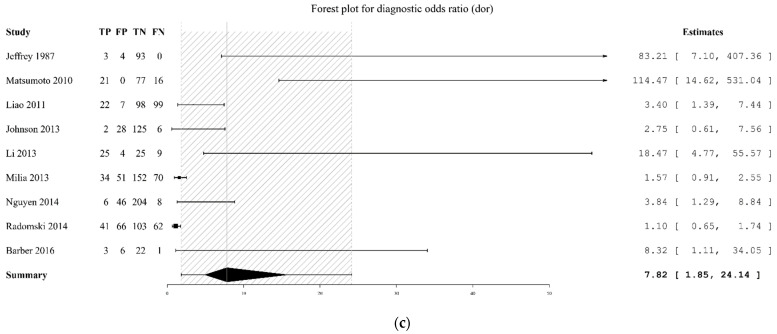
Forest plot of sensitivity (**a**), specificity (**b**), and diagnostic odds ratio (**c**) for shock development after computed tomography. The values of true positive (TP), false positive (FP), true negative (TN), and false negative (FN) are shown. The model-based mean estimates with 95% credible intervals are also shown [15,18,19,20,21,22,23,24,26].

**Figure 4:**
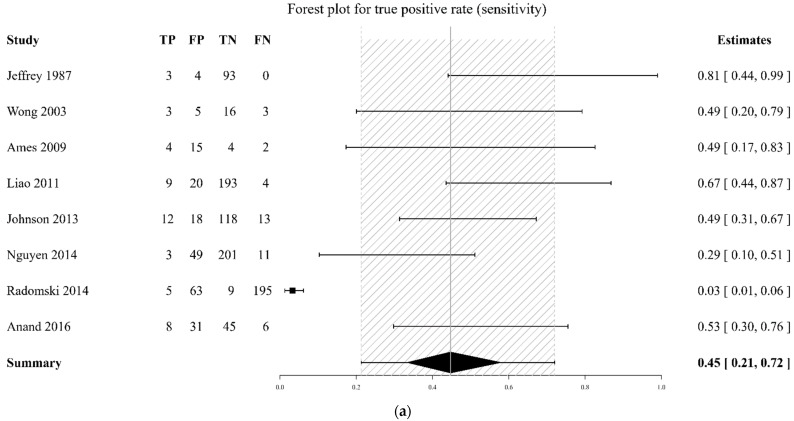

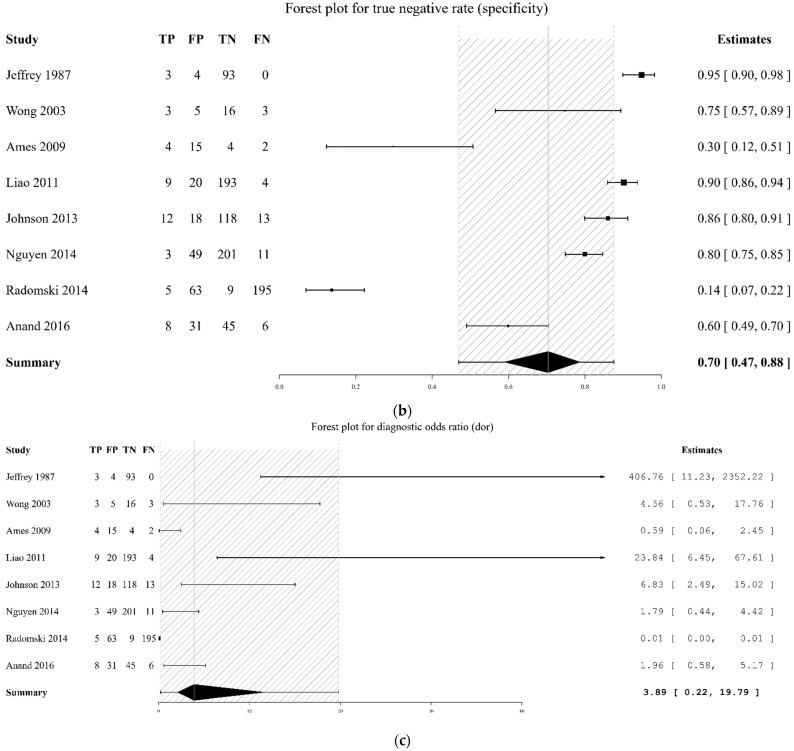
Forest plot of sensitivity (**a**), specificity (**b**), and diagnostic odds ratio (**c**) for mortality after computed tomography. The values of true positive (TP), false positive (FP), true negative (TN), and false negative (FN) are shown. The model-based mean estimates with 95% credible intervals are also shown [15,16,17,19,20,23,24,25].

**Figure 5:**
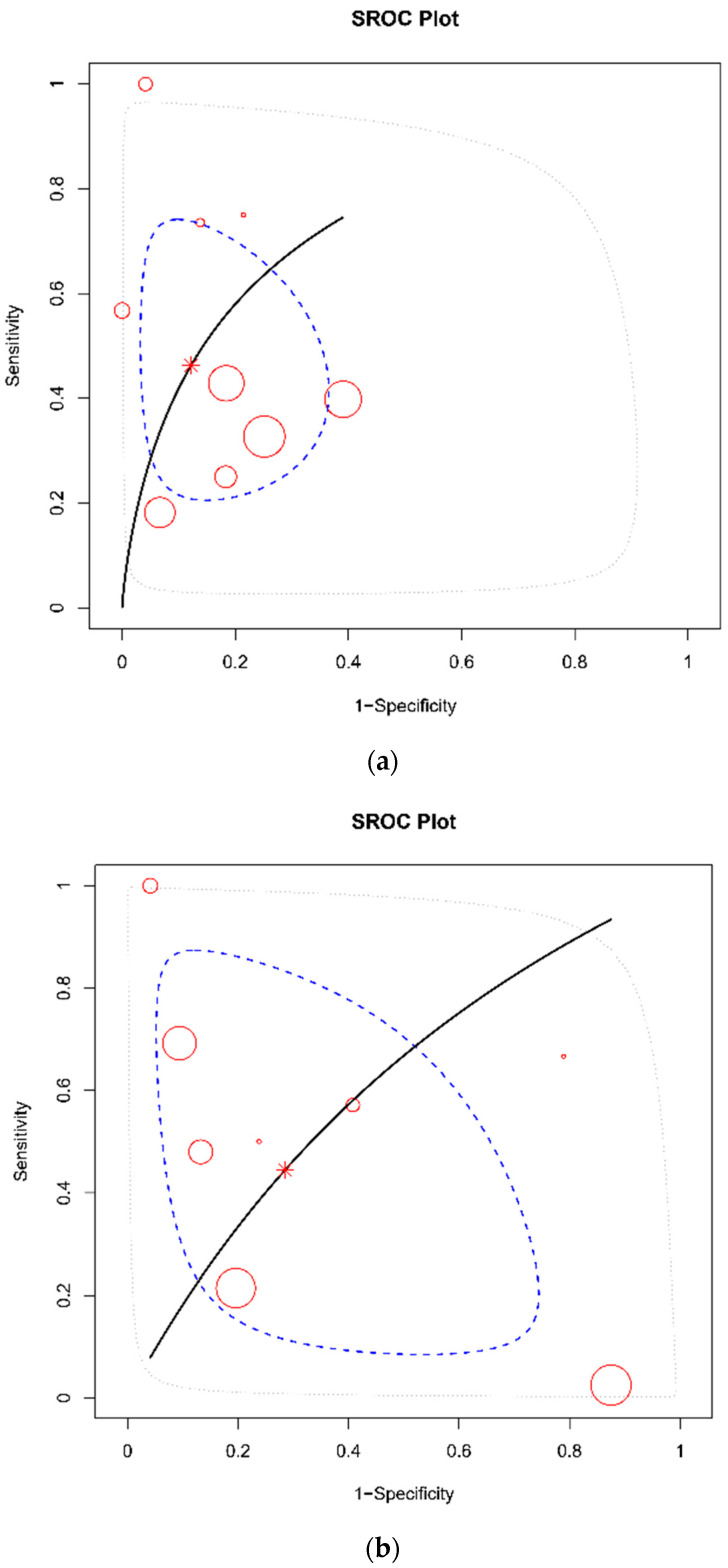
SROC plot for shock development (**a**) and mortality (**b**). Each bubble represents one study and indicates its observed sensitivity and specificity. The size of the bubble is proportional to the number of individuals in the study. The solid black line is the SROC line. The star point represents the summary estimate. The dashed blue line is the 95% credible region and the dashed gray is the 95% prediction region.

The DTA of the included studies was summarized in Figure 3 and Figure 4 and Table 2. The pooled sensitivity of a flat IVC for the development of shock was 0.46 (95% CrI 0.32–0.63), while the pooled specificity was 0.87 (95% CrI 0.78–0.94, Figure 2). The pooled diagnostic odds ratio (DOR) for shock was 7.74 (95% CrI 1.82–23.85). The pooled sensitivity of a flat IVC for mortality was 0.45 (95% CrI 0.21–0.72), while the pooled specificity was 0.70 (95% CrI 0.47–0.88, Figure 3). The pooled DOR for mortality was 3.89 (95% CrI 0.22–19.79). The AUC of a flat IVC for shock and morality were 0.78 (95% CrI 0.58–0.93, I2 = 41.65%) and 0.60 (95% CrI 0.26–0.89, I2 = 0%), respectively (Table 2). The SROC with prediction and credible region is depicted in Figure 5.

### 3.4. Subgroup Analysis, Sensitivity Analysis, and Evaluation of Heterogeneity

**Figure 6:**
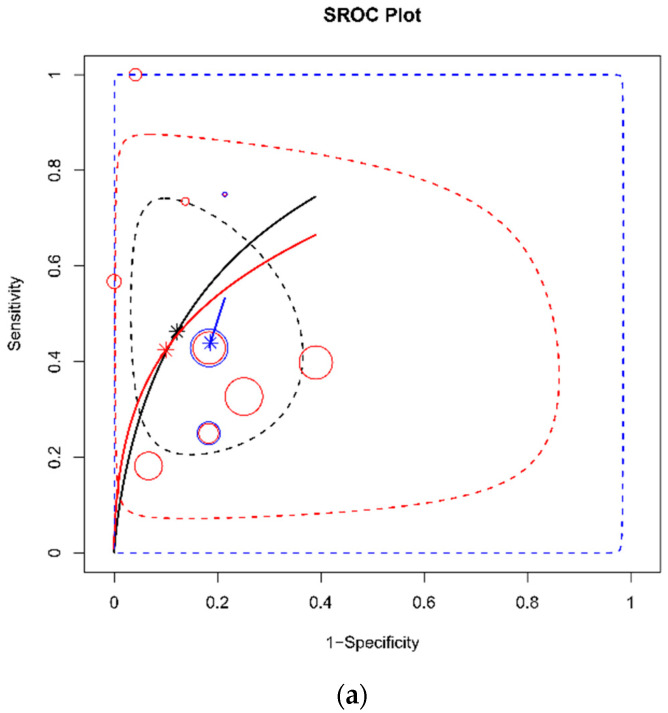

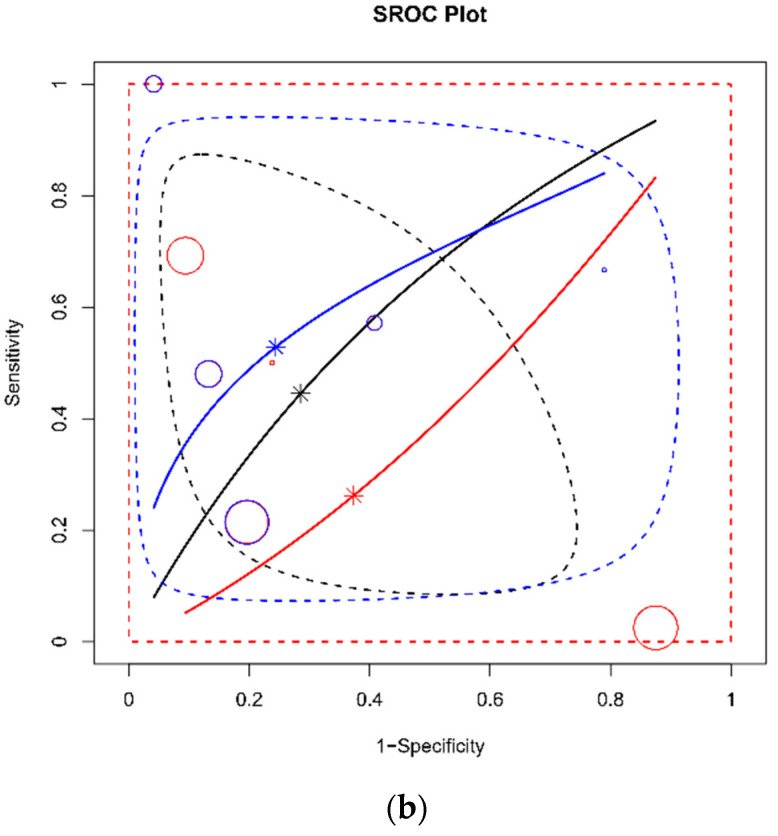
SROC plot to compare the result of each sub-analysis for shock development (**a**) and mortality (**b**). In the sub-analyses, for shock development (**a**), the black color represents the result of overall data, the red represents the result of data from the flat inferior vena cava (IVC) ratio ≥ 3, and the blue color represent the result of data from others (1.9 ≤ flat IVC ratio ≤ 2). In sub-analysis for mortality (**b**), the black color represents the result of overall data, the red represents the result of data from flat IVC ratio ≥ 3, and the blue color represent the result of data from others (1.9 ≤ flat IVC ratio ≤ 2 or not-reported). Each bubble represents one study and indicates its observed sensitivity and specificity. The size of the bubble is proportional to the number of individuals in the study. The solid line is the SROC line. The star point represents the summary estimate. The dashed line is the 95% credible region.

The subgroup analysis and sensitivity analysis were conducted and summarized in Table 2. The threshold of a flat IVC and risk of bias were considered possible confounders. In terms of the DTA for shock, the lower threshold (T/AP ratio between 1.9 and 2.5) [20,23,26] showed a lower AUC (0.58, 95% CrI 0.19–0.87), while the higher threshold (T/AP ratio ≥ 3) [18,19,21,22,24] showed a similar AUC to the overall group (0.80 AUC vs. 0.79 AUC). In the group with a high risk of bias [15,22,26], the AUC was similar to overall group (0.79 AUC). In terms of the measuring site of IVC, both infrahepatic [15,19,21,26] and renal levels [18,20,22,23,24] showed similar AUCs (0.78 vs. 0.80). In terms of the DTA for mortality, the AUC was substantially lower in the high threshold (T/AP ratio ≥ 3, 0.43 AUC, 95% CrI 0.03–0.93) [16,19,24] and in the low or unclear risk of bias group (0.48 AUC, 95% CrI 0.13–0.83) [15,17,20,23,25]. For mortality, the IVC measured at the renal level [16,20,23,24,25] showed a poor AUC of 0.44. The result of each sub-analysis for shock development and mortality is depicted in Figure 6. The sub-analysis for mortality showed substantial heterogeneity compared to shock development. In the test for threshold effect, the Spearman’s rank correlation rho was 0.23 (*p* = 0.23) in the DTA for shock was 0.54 (*p* = 0.17).

### 3.5. Publication Bias

**Figure 7:**
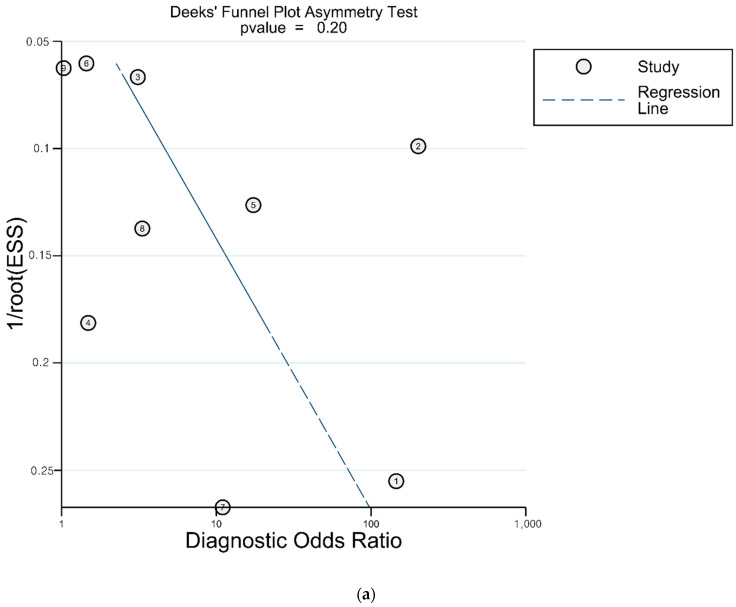

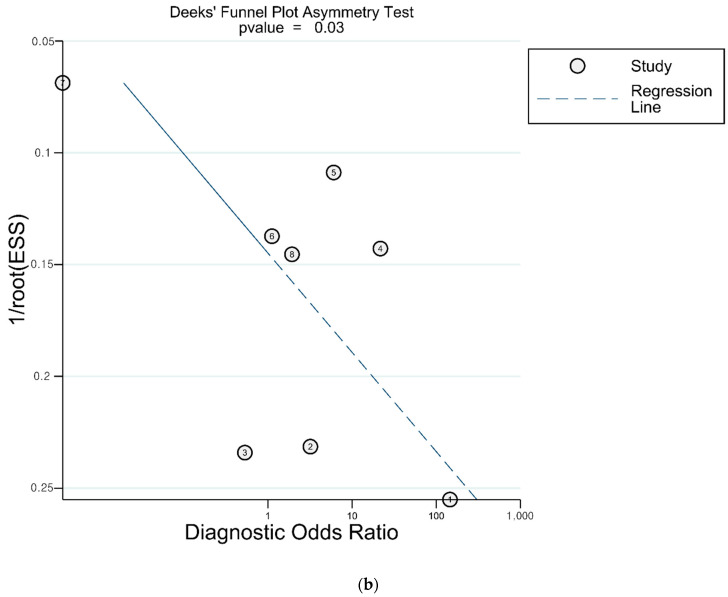
Asymmetry test for publications bias. (**a**) The diagnostic test accuracy for possible shock (**b**) and the diagnostic test accuracy for mortality.

In terms of the DTA for development of shock, there was no asymmetry on visual inspection in Deek’s funnel plot, and there was no statistically significant asymmetry (*p* = 0.20). However, in terms of the DTA for mortality, there was a significant asymmetry (*p* = 0.03) (Figure 7).

## 4. Discussion

In our meta-analysis of 12 studies with a total of 1706 trauma patients, the flat IVC showed acceptable diagnostic accuracy to predict the development of shock with an AUC of 0.78. In contrast, the flat IVC showed poor accuracy for morality prediction with an AUC of 0.60. The high ratio of flat IVCs (T/AP ratio ≥ 3) also showed high accuracy with an AUC of 0.80. However, the pooled sensitivity of a flat IVC for the development of shock was very low (0.46), while the pooled specificity was 0.87. Using CT, the detecting power of shock may be poor, but the high specificity would be useful to rule out the shock. However, our results should be interpreted with caution, and careful clinical application is warranted due to the high risk of bias and substantial heterogeneity across the studies. More well-designed studies are needed to estimate the true effect size. Nonetheless, to the best of our knowledge, this is the first meta-analysis that reports the quantitative pooled diagnostic test accuracy of a flat IVC on CT in trauma patients.

Even the expert trauma surgeon could not predict the hypovolemia accurately. Data from a larger multicenter trial from ten level 1 trauma centers in the United States demonstrated that the clinical gestalt to predict the need for a massive transfusion showed 65.6% sensitivity, 63.8% specificity, and 0.63 AUC [27]. Likewise, accuracies of ABC (0.64 AUC) and TASH scores (0.72 AUC) were not high in the prospective study [27]. To achieve damage control resuscitation, hemostasis and resuscitation should not be delayed [28]. Vitals might not be altered until there is a substantial volume loss because the compensatory mechanism that responds to intravascular volume depletion might remain intact [3]. Thus, the flat IVC on CT could be a useful tool.

Recently, Elst et al. conducted a systematic review of the signs of post-traumatic hypovolemia on abdominal CT [7]. The authors investigated the hypovolemic shock complex comprising flat IVCs, IVC halo, aortic diameter, shock bowel, heterogeneous parenchymal enhancement of liver, pancreas enhancement, peripancreatic fluid, adrenal enhancement, kidney enhancement, spleen volume change, spleen enhancement, and gall bladder enhancement. The authors reported that a flat IVC was one of the most frequent CT signs of hypovolemia and had the highest predictive value for hypovolemia. However, the authors did not conduct a meta-analysis. Our previous meta-analysis demonstrated that ultrasound-guided measurement of the variability of IVC diameter had high accuracy (AUC of 0.86) for volume responsibility [5]. The diameter of the IVC varies with inspiration and expiration and reflects cardiac preload. This variation can be measured using ultrasound. However, in most studies included in this previous meta-analysis, ultrasounds were performed by experienced cardiologists or intensivists. In general, the quality of ultrasound measurement depends on the operator. Furthermore, the IVC in ultrasounds can be invisible in patients with obesity, intra-abdominal fluid collection, and high bowel gas. Contrastively, CT provides objective images to clinicians. Moreover, various levels of the IVC can be measured in a CT image. In our study, the most frequent measure site was at the renal vein and infrahepatic levels. However, CT is not recommended for patients with hemodynamic instability [3]. In our meta-analysis, patients with hemodynamic stability underwent CT in six included studies. Therefore, CT may be useful for initially stable trauma patients.

In general, during CT, patients are recommended to pause their breathing after full inspiration to enhance the image quality of chest CT, which is usually performed simultaneously with abdominal CT. During inspiration, thoracic pressure decreases, and venous return increases [5]. Consequently, IVC diameter decreases during inspiration in patients with spontaneous breathing [5]. Therefore, IVC diameter on CT may be representative of the minimum diameter of the IVC. Indeed, IVC diameter on CT is a static measurement compared to the IVC on an ultrasound. Interestingly, a retrospective study including 64 euvolemic outpatients reported six (10%) patients with a flat infrahepatic IVC in a pre-contrast scan [29]. However, a post-contrast scan of these six patients showed a more distended IVC. In this study, all patients were requested to fast from midnight on the eve. Thus, this fasting may have induced hypovolemia. Nonetheless, clinicians should not prejudge hypovolemia when a flat IVC on CT is observed since the sensitivity was very low in the present review. Indeed, the IVC diameter on CT is a snapshot and it can be changed according to hemodynamic status. Thus, additional point-of-care measurement such as ultrasonography would be useful for detect the change.

In our meta-analysis, substantial heterogeneity in some eligible studies such as measure site of IVC or patient’s age. To evaluate the influence of heterogeneity, we conducted a subgroup analysis. In our subgroup analysis, studies with a high ratio of flat IVCs (T/AP ratio ≥ 3) showed a slightly higher pooled AUC of 0.80 for possible shock, while studies with a T/AP ratio between 1.9 and 2.5 showed a lower pooled AUC of 0.58. Therefore, a high ratio of flat IVCs appears more useful. The subgroup analysis of the risk of bias showed similar results. In terms of the measuring site of the IVC, both infrahepatic and renal levels appear to be useful. In terms of mortality, the subgroup analysis showed substantial heterogeneity. Moreover, the publication bias test showed significant asymmetry in terms of mortality. Thus, further studies are needed, and the diagnostic accuracy of a flat IVC for morality appears not to be confidential.

In our systematic review, we found other outcomes, such as anemia and massive transfusion. Two retrospective studies reported that IVC diameter was significantly lower in an anemia group [30,31]. However, these studies reported only IVC diameter, not the flat ratio. Thus, we excluded these studies. The prediction of anemia using IVC diameter may be a potential outcome for future study. Three retrospective studies reported a massive transfusion requirement as a primary outcome [32,33,34]. Akasaki et al. reported that a flat IVC with a T/AP ratio ≥ 3 was a significant risk factor for massive transfusion in a multivariable logistic regression [32]. Chien et al. reported that IVC volume was significantly related to massive transfusion in a multivariable logistic regression [33]. Takada et al. reported that IVC diameter was significantly related to massive transfusion in a multivariable logistic regression [34]. Due to insufficient information for diagnostic test accuracy, we excluded these three studies. However, we noted the potential of the IVC to predict massive transfusion. The triggering of massive transfusion is a crucial issue in severe trauma patients, and future studies are needed to evaluate the relationship between the IVC and massive transfusion.

Our study had several limitations. First, all the studies included were observational. Second, there was substantial heterogeneity across the studies. Several studies had a high risk of bias in terms of patient selection and an unclear risk of bias in terms of flow and timing. Third, the threshold of the index tests varied, and there was considerable heterogeneity. To overcome this issue, we investigated the correlation between sensitivity and specificity to detect the threshold effect and conducted a subgroup analysis. Fourth, the small number of studies included in this review may cause statistical instability of the model. To enhance the model stability, we used Bayesian statistics. Fifth, the sensitivity of a flat IVC was too low. However, the high specificity suggests that a flat IVC may be useful to rule out the volume depletion. Six, Deek’s funnel plot showed significant publication bias regarding mortality and should be considered when interpreting our results. This may be due to small study effect. The DTA for mortality appears limited. Seventh, the measurement site of the IVC was heterogeneous. The most common site was at the renal vein level, which was reported in seven studies. We conducted a subgroup analysis to overcome this issue. Eighth, six included studies did not reported vitals during CT. However, we hypothesized that patients in these studies would be stable because the CT is not recommended for unstable patients in clinical guideline. Ninth, there was no clear description regarding the type of shock (hypovolemic or septic) in eligible studies in our review except one study [21]. However, one study reported that the septic shock of non-trauma patients was significantly related to the increased IVC ratio [35]. Further future study is warranted. Finally, we included only published original articles and those written in English.

## 5. Conclusions

Our systematic review and meta-analysis suggest that a flat IVC in trauma patients on CT, in terms of the development of shock, provides acceptable diagnostic accuracy with high specificity even with low sensitivity. In terms of in-hospital mortality, a flat IVC showed low accuracy. However, a high risk of bias and substantial heterogeneity in the included studies limit the generalization of our results. Clinicians should exercise caution when using this modality. To determine the exact effect size, a further large-scale prospective study is warranted.

## Figures and Tables

**Table 1:** Main characteristics of the eligible studies.

Author	Year	Study Type	Study Period	Location	Outcome	Patients	Index	Measure Site	CT Timing	Vitals during CT Scan	CT Slice	Threshold of Flat IVC Ratio
Jeffrey [15]	1987	retrospective	January–June 1987	San Francisco, USA	hypotension (SBP < 100mmHg), mortality	100 patients with abdominal trauma	flat IVC	infrahepatic level	NR	NR	1	NR
Wong [16]	2003	retrospective	1996–2000	Taiwan	early intervention, mortality	32 BAT patients with contrast extravasation	flat IVC	renal vein level	within 3 h from admission	stable	1	T/AP ratio = 4
Ames [17]	2009	retrospective	2006–2008	Pittsburgh, USA	mortality	25 trauma patients	flat IVC	at least three contiguous sections	NR	NR	4 to 64 MDCT	AP diameter = 9 mm
Matsumoto [18]	2010	retrospective	2005—2007	3 hospitals, Japan	shock (SBP <90 mmHg or hear rate > 120 beats per min)	114 adult patients with blunt torso trauma	flat IVC	renal vein level	within 30 min from admission	stable	16 or 64 MDCT	T/AP ratio = 4
Liao [19]	2011	retrospective	2003–2006	Taiwan	shock (SBP < 100 mmHg), mortality	226 patients with blunt trauma (liver, spleen kidney)	flat IVC	infrahepatic level	within 1 h from admission	stable	4	TAP ratio = 3, AP diameter = 9 mm
Johnson [20]	2013	retrospective	Jan–Dec 2010	Oklahoma, USA	shock (SBP <90 mmHg), mortality	161 Trauma patients with ISS ≥ 9, ≥16 year	flat IVC	renal vein level	within 1 h from admission	stable	NR	T/AP ratio = 1.9
Li [21]	2013	retrospective	2008–2011	China	hypovolemic shock (SBP < 90 mmHg and HR > 120 beats/min with urine output <30mL/h or lactate level < 2mmol/L within 24 hrs.	63 adult trauma patients with multiple injuries	flat IVC	infrahepatic level	within 2 h from admission	stable	64 MDCT	T/AP ratio = 3.02
Milia [22]	2013	retrospective	2006–2011	Wisconsin, USA	shock (adjusted shock index > 50)	307 elderly (≥55 years) patients with ISS ≥ 15	flat IVC	renal vein level	within 1 h from admission	NR	64 MDCT	T/AP ratio = 3
Nguyen [23]	2014	retrospective	2012–2013	San Diego, USA	ED hypotension (≤90 mmHg), mortality	264 adult patients with major trauma activation	flat IVC	renal vein level	within 1 h from admission	NR	64 MDCT	T/AP ratio = 2.5
Radomski [24]	2014	retrospective	January–December 2012	Washington, USA	shock (shock index > 0.7), mortality	272 adult who met highest-level trauma activation criteria	flat IVC	renal vein level	within 30 min from admission	stable	64 MDCT	T/AP ratio = 3 (shock), 1.9 (mortality)
Anand [25]	2016	retrospective	2010–2011	Bakersfield, USA	mortality, MT	90 trauma patients	flat IVC	renal vein level	within 1 h from admission	NR	NR	AP diameter = 9 mm
Barber [26]	2016	retrospective	2012–2013	London, UK	development of shock (hypotension and tachycardia using the PALS reference range)	52 pediatric trauma patients (<16 years)	flat IVC	infrahepatic level	NR	NR	128 MDCT	T/AP ratio = 2

SBP, systolic blood pressure; CT, computed tomography; IVC, inferior vena cava; NR, not reported; BAT, blunt abdominal trauma; T/AP, transverse/anteroposterior; MDCT, multi-detector computed tomography; ISS, injury severity score; APLS, advanced pediatric life support.

**Table 2:** Subgroup and sensitivity analysis of diagnostic test accuracy of flat IVC.

Subgroup	Pooled Sens (95% CrI)	Pooled Spec (95% CrI)	Pooled LRpos (95% CrI)	Pooled LRneg (95% CrI)	Pooled DOR (95% CrI)	AUC (95% CrI)	I2	Cochran’s Q (*p*)
Flat IVC for Shock (overall) (k = 9) [15,18,19,20,21,22,23,24,26]	0.46 (0.32–0.63)	0.87 (0.78–0.94)	4.24 (1.51–10.51)	0.62 (0.37–0.86)	7.70 (1.77–24.04)	0.78 (0.58–0.93)	41.7%	13.71 (*p* = 0.090)
for Shock (threshold, T/AP ratio, 3 or more) (k = 5) [18,19,21,22,24]	0.43 (0.24–0.64)	0.88 (0.68–0.98)	7.32 (0.86–33.31)	0.67 (0.36–1.07)	14.73 (0.79–72.55)	0.80 (0.47–0.98)	56.1%	9.11 (*p* = 0.058)
for Shock (threshold, T/AP ratio between 1.9 and 2.5) (k = 3) [20,23,26]	0.44 (0.18–0.75)	0.81 (0.73–0.87)	2.47 (0.76–5.04)	0.68 (0.24–1.07)	5.11 (0.71–19.37)	0.58 (0.19–0.87)	0%	1.81 (*p* = 0.404)
for Shock (high risk of bias) (k = 3) [15,22,26]	0.65 (0.27–0.98)	0.85 (0.66–0.96)	6.88 (0.77–25.06)	0.44 (0.01–1.12)	430.02 (0.66–1420.98)	0.79 (0.33–0.99)	9.8%	2.22 (*p* = 0.330)
for Shock (low or unclear risk of bias) (k = 6) [18,19,20,21,23,24]	0.42 (0.25–0.61)	0.88 (0.73–0.97)	5.08 (1.06–18.31)	0.67 (0.40–0.98)	9.01 (1.09–37.91)	0.79 (0.52–0.97)	32.5%	7.41 (*p* = 0.192)
for Shock (measuring site = infrahepatic) (k = 4) [15,19,21,26]	0.62 (0.27–0.93)	0.91 (0.81–0.96)	7.85 (1.75–20.71)	0.43 (0.05–0.88)	53.17 (2.12–282.45)	0.78 (0.36–0.98)	6.5%	3.21 (*p* = 0.36)
for Shock (measuring site = renal) (k = 5) [18,20,22,23,24]	0.41 (0.30–0.51)	0.84 (0.63–0.97)	5.14 (0.75–24.37)	0.73 (0.50–1.20)	8.7 (0.63–45.13)	0.80 (0.49–0.98)	56.80%	9.27 (*p* = 0.055)
Flat IVC for Mortality (overall) (k = 8) [15,16,17,19,20,23,24,25]	0.45 (0.21–0.72)	0.70 (0.47–0.88)	1.96 (0.38–6.04)	0.85 (0.29–1.75)	3.96 (0.22–19.78)	0.60 (0.26–0.89)	0%	6.624 (*p* = 0.469)
for Mortality (threshold, T/AP ratio, 3 or more) (k = 3) [16,19,24]	0.30 (0.05–0.72)	0.61 (0.23–0.91)	1.77 (0.05–9.54)	1.59 (0.25–5.46)	8.51 (0.01–36.63)	0.43 (0.03–0.93)	0%	1.552 (*p* = 0.460)
for Mortality (threshold, T/AP ratio between 1.9 and 2.5 or IVC diameter under 9mm) (k = 5) [15,17,20,23,25]	0.52 (0.32–0.78)	0.74 (0.45–0.92)	2.84 (0.68–9.24)	0.69 (0.23–1.45)	6.41 (0.46–31.17)	0.71 (0.34–0.94)	38.1%	6.463 (*p* = 0.167)
for Mortality (low or unclear risk of bias) (k = 6) [17,19,20,23,24,25]	0.36 (0.14–0.65)	0.61 (0.32–0.84)	1.24 (0.19–4.25)	1.19 (0.41–2.91)	1.89 (0.07–9.81)	0.48 (0.13–0.83)	0%	4.343 (*p* = 0.501)
for Mortality (measuring site = renal) (k = 5) [16,20,23,24,25]	0.28 (0.09–0.56)	0.64 (0.36–0.85)	1.12 (0.14–4.37)	1.24 (0.45–2.68)	1.71 (0.05–9.54)	0.44 (0.11–0.83)	0%	3.428 (*p* = 0.489)

IVC, inferior vena cava; CrI, credible interval; Sens, sensitivity; Spec, specificity; LRpos, positive likelihood ratio; LRneg, negative likelihood ratio; DOR, diagnostic odds ratio; AUC, area under the curve; T/AP, transverse/anteroposterior; infrahepatic, infrahepatic IVC; renal, renal IVC.

## Data Availability

Not applicable.
